# *Phoenix dactylifera* (Ajwa Dates) Alleviate LPS-Induced Sickness Behaviour in Rats by Attenuating Proinflammatory Cytokines and Oxidative Stress in the Brain

**DOI:** 10.3390/ijms241310413

**Published:** 2023-06-21

**Authors:** Thippeswamy Boreddy Shivanandappa, Ghallab Alotaibi, Maheswari Chinnadhurai, Sudharshan Reddy Dachani, Mahmad Dabeer Ahmad, Khalid Abdullah Aldaajanii

**Affiliations:** 1 Department of Biomedical Science, College of Pharmacy, Shaqra University, Al-Dawadmi Campus, Al-Dawadmi 11961, Saudi Arabia; khaledaldaajanii@gmail.com; 2 Department of Pharmaceutical Sciences, College of Pharmacy, Shaqra University, Al-Dawadmi Campus, Al-Dawadmi 11961, Saudi Arabia; ghalotaibi@su.edu.sa (G.A.); mahmad@su.edu.sa (M.D.A.); 3 Department of Pharmacy Practice, College of Pharmacy, Shaqra University, Al-Dawadmi Campus, Al-Dawadmi 11961, Saudi Arabia; maki3kp@gmail.com (M.C.); suhasinraydaa@su.edu.sa (S.R.D.)

**Keywords:** Ajwa date fruits, sickness behaviour, antioxidants, quercetin, cytokines

## Abstract

Traditional medicine claims that various components of the *Phoenix dactylifera* (date plant) can be used to treat memory loss, fever, inflammation, loss of consciousness, and nerve disorders. The present study aims to evaluate the effectiveness of *Phoenix dactylifera* fruit extracts (PDF) against rat sickness behaviour caused by lipopolysaccharide (LPS) by assessing behavioural and biochemical parameters. PDF was prepared by extracting dry fruits of *P. dactylifera* with a methanol:water (4:1, *v*/*v*) mixture. The PDF was evaluated for phenolic and flavonoid content and HPLC analysis of quercetin estimation. Adult Wistar rats were treated with LPS, PDF + LPS and dexamethasone + LPS. Water and food intake, behavioural tests such as locomotor activity, tail suspension and forced swim tests were conducted. Furthermore, alanine transaminase (ALT) and aspartate transaminase (AST) were estimated in plasma and malondialdehyde (MDA), reduced glutathione (GSH), nitrite, tumor necrosis factor-α (TNF-α) and interleukin-6 (IL-6), were estimated in the brain. PDF ameliorated LPS-induced sickness behaviour by reducing MDA, nitrite, IL-6, and TNF-α levels and improving GSH, behavioural alteration, water and food intake in the treated rats. In the plasma of the treated rats, PDF also decreased the levels of ALT and AST. The outcomes demonstrated the efficacy of PDF in reducing the sickness behaviour caused by LPS in rats. The authors believe that this study will provide the groundwork for future research to better understand the underlying mechanisms of action and therapeutic efficacy.

## 1. Introduction

Dates (*Phoenix dactylifera* L., family—Arecaceae) are widely grown as a food and commercial crop in countries including India and Pakistan [1]. Ajwa dates are a unique variety of date fruit grown in Al-Madina Al-Munawwarah, Saudi Arabia and they have medicinal properties [1,2]. The carb-rich Ajwa date is a fantastic source of proteins, vitamins, high dietary fibre, minerals, and lipids [3]. They contain typical minerals such as iron, copper, zinc, calcium, potassium, cobalt, fluorine, sulphur, magnesium, manganese, phosphorus, selenium, and boron [2]. A variety of phytochemicals of Ajwa date include glycosides, polyphenols, flavonoids, and sterols. Ajwa dates have hepatoprotective [4], cardioprotective [5], nephroprotective [6], antioxidant [7,8], antihyperlipidemic [7], anti-inflammatory [8], antibacterial [2] and anti-cancer [1] properties. 

In the Middle East, dates have been used as a staple meal for thousands of years [9]. Various religions give high importance to date fruits [10]. In Islam, to break the daylong fast during the holy month of Ramadan, dates are used [10]. Date palms are the best assets, according to the Prophet Muhammad, who also advised consuming dates and developing a preference for date palms [11]. Jews celebrate Palm Sunday and consider dates to be one of the seven holy fruits [11]. Many people in the Middle East believe that eating date fruits on an empty stomach can counteract the poisonous effects of any substances to which the individual may have been exposed [6]. In traditional medicine, the date plant is used to treat fever, inflammation, paralysis, nerve disorders, and memory loss [2,6]. All these overlap with the signs and symptoms of sickness behaviour [12]. The impact of dates on the CNS is extensively documented [6], on the other hand, there are no reports of its advantageous benefits on sickness behaviour.

A sophisticated and coordinated adaptive change brought on by tissue damage or acute infections is known as sickness behaviour [13,14,15]. The sickness behavioural pattern includes malaise, hyperalgesia, fever, lethargy, social retreat, inhibition, decreased locomotor activity, exploration, grooming, loss of libido, anhedonia, sleepiness, anorexia weight loss, disrupted concentration, and anxiety [14,16]. Even while sick behaviour is a normal immune response for faster recovery from an infection or damage, it nonetheless causes discomfort in the victims if it lasts for a longer period [12]. So, it is important to address sickness behaviour to reverse the patients’ altered social, cognitive, and mental functioning. 

The effect of medications on sickness behaviour is preclinically evaluated using a variety of animal models. Preclinical research uses lipopolysaccharide (LPS)-induced sickness behaviour in rodents the most frequently among others [12,17]. 

Based on the aforementioned information, the current investigation was carried out to determine the effectiveness of date fruit extracts against LPS-induced illness behaviour in rats by evaluating behavioural and biochemical markers.

## 2. Results

### 2.1. Standardisation of PDF

Total phenolic content in PDF was found to be 5.57 ± 0.31 mg/g of gallic acid equivalent weight (mean ± SEM, n = 3) and total flavonoid content was found to be 171.2 ± 1.15 mg/g of quercetin equivalent weight (mean ± SEM, n = 3). 

**Figure 1:**
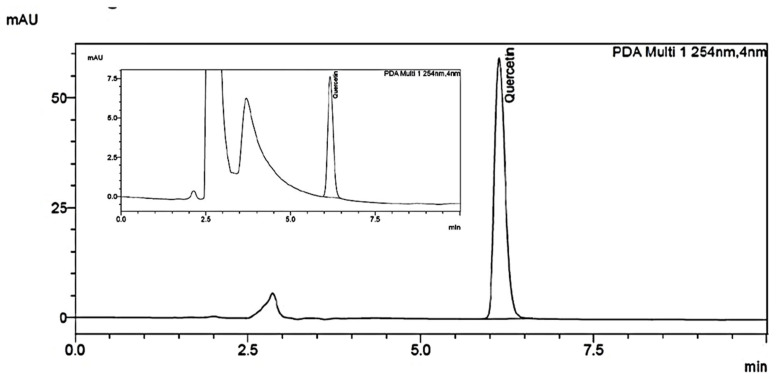
HPLC Chromatogram of PDF showing the presence of Quercetin (Inset), separated on a Waters xbridge ^®^ RP C18 column (5 µm, 4.6 × 250 mm) using isocratic elution was performed using 70% methanol, 30% milli-q water and 0.1% trifluoro acetic acid at a total flow rate of 1.0 mL/min with a run time of 10 min. The chromatogram at 254 nm was analysed.

The HPLC fingerprint profile of PDF was standardised with biomarker and it served as a standard for comparison in the subsequent preparation of PDF. HPLC chromatogram of the extract was found to contain constituents eluting between 2.0 min and 7.0 min. The presence of Quercetin in PDF at RT 6.171 min was confirmed by comparing its retention time and UV spectra with that of the standard Quercetin. The amount of Quercetin present in PDF was found to be 56.9 µg/mg. HPLC Chromatogram of PDF and quercetin is shown in Figure 1.

### 2.2. Effect of PDF on Water and Food Intake in LPS-Treated Rats

Treatment with LPS leads to reduced water and food intake in the rats by 63.77% and 55.66%, respectively, when compared to the vehicle control group. Administration of PDF 250 and PDF 500 mg/kg to LPS-challenged animals restored the water and food intake by 84.04% and 134.66%, respectively, when compared to the LPS control animals. Animals administered with PDF 500 mg/kg (15.3 ± 0.56 and 4.66 ± 0.33, respectively) consumed almost the same amount of water and food as animals treated with 1 mg/kg of dexamethasone (15.6 ± 0.59 and 4.83 ± 0.40, respectively). The effect of PDF on water and food intake in LPS-treated rats is shown in Table 1. 

### 2.3. Effect of PDF on Behavioural Outcomes

#### 2.3.1. Locomotor Activity Scores

**Figure 2:**
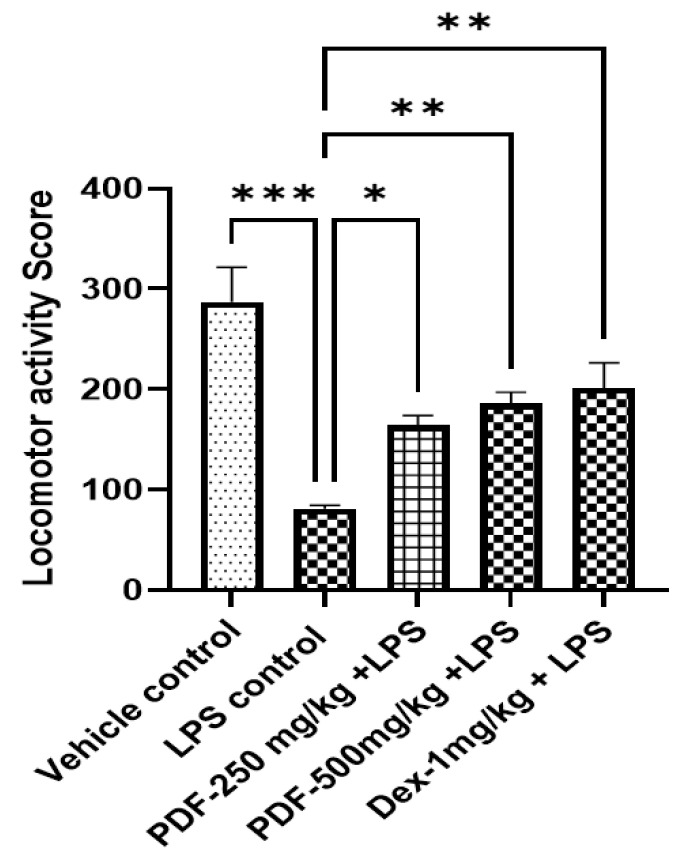
Effect of *Phoenix dactylifera* fruit extracts on locomotor activity score in actophotometer test of lipopolysaccharide treated rats. Numerical values are represented as mean ± SEM (n = 6), * *p* < 0.05, ** *p* < 0.01 and *** *p* < 0.001.

Compared to the vehicle control group, the locomotor score in LPS control rats was drastically decreased (*p* < 0.001). Treatment with both doses of PDF improved (*p* < 0.05 and *p* < 0.01, respectively) the locomotor score in LPS-treated rats when compared to LPS control rats. The improvement in locomotor score in a higher dose of PDF was comparable with the scores of the standard drug dexamethasone (*p* < 0.001). The effect of PDF on locomotor score in LPS-treated rats is shown in Figure 2. 

#### 2.3.2. Tail Suspension Test

**Figure 3:**
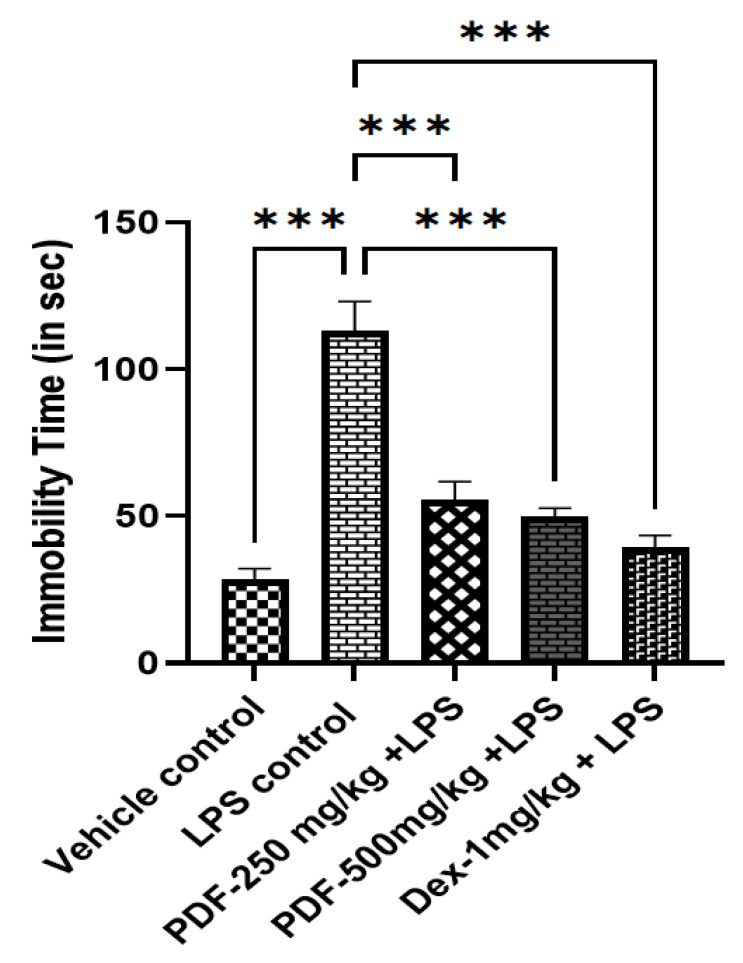
Effect of *Phoenix dactylifera* fruit extracts on tail suspension test induced-immobility in lipopolysaccharide treated rats. Numerical values are represented as mean ± SEM (n = 6), *** *p* < 0.001.

In the tail suspension test, compared to the vehicle control rats LPS control rats had significantly (*p* < 0.001) higher periods of immobility. Treatment of both the doses of PDF and the standard drug dexamethasone significantly reduced the immobility in LPS-treated rats when compared to the LPS-control rats. The effect of PDF on tail suspension test-induced immobility in LPS-treated rats is shown in Figure 3. 

#### 2.3.3. Despair Behaviour Test

**Figure 4:**
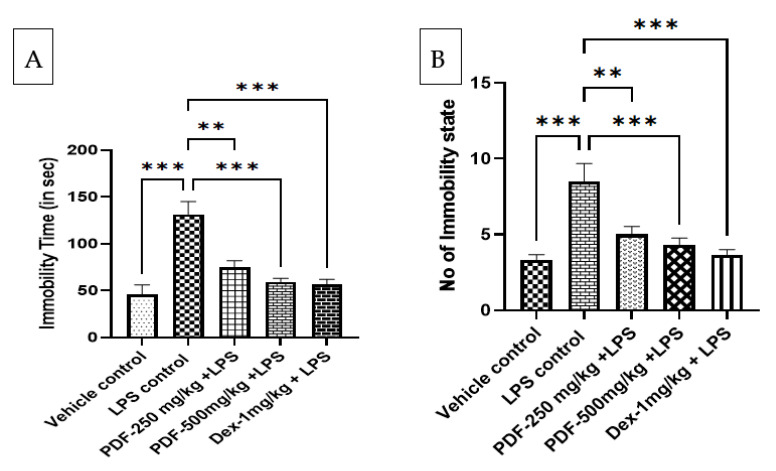
Effect of *Phoenix dactylifera* fruit extracts on forced swim test-induced immobility in lipopolysaccharide treated rats, (**A**) Immobility time and (**B**) Number of immobility state. Numerical values are represented as mean ± SEM (n = 6), ** *p* < 0.01 and *** *p* < 0.001.

The despair behaviour of rats was measured by the forced swim-induced immobility in the rats. Compared to the vehicle control rats the immobility time and the number of immobility states are significantly (*p* < 0.001) greater in LPS-control rats. Treatment with PDF at the dose of 250 mg/kg and 500 mg/kg drastically (*p* < 0.01 and *p* < 0.001, respectively) reduced the immobility time and number of immobility states in LPS-treated rats. The higher dose of PDF administration gives almost equivalent results to that of the standard control drug dexamethasone (*p* < 0.001). The results of the effect of PDF on forced swim test-induced immobility in LPS-treated rats are shown in Figure 4. 

### 2.4. Effect of PDF on ALT and AST Levels in Serum

**Figure 5:**
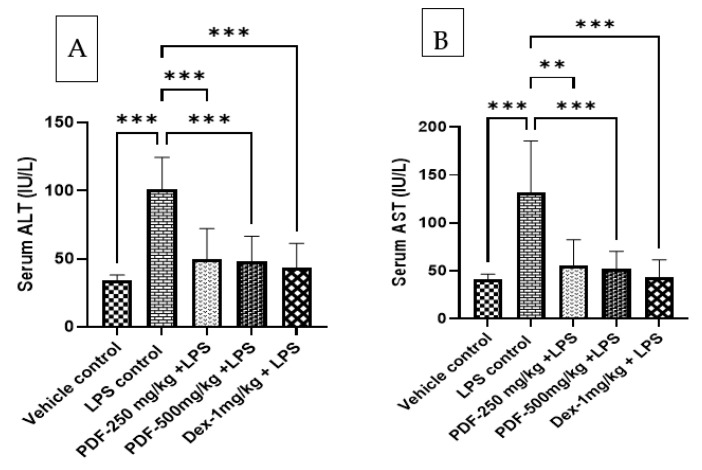
Effect of *Phoenix dactylifera* fruit extracts on serum (**A**) Alanine transaminase and (**B**) Aspartate transaminase levels in lipopolysaccharide treated rats. Numerical values are represented as mean ± SEM (n = 6), ** *p* < 0.01 and *** *p* < 0.001.

The administration of LPS elevated the levels of liver marker enzyme ALT and AST in the serum when compared to the vehicle control rats and the values are found to be statistically significant with a *p*-value less than 0.001. The administration of PDF to LPS-treated rats significantly reduced the levels of ALT and AST when compared to the LPS control rats. The result of PDF-treated rats is comparable with the dexamethasone (*p* < 0.001) treated rats. The results of the effect of PDF on serum ALT and AST levels are shown in Figure 5.

### 2.5. Tissue Biochemical Parameters

#### 2.5.1. Effect of PDF on Oxidative and Nitrative Stress

**Figure 6:**
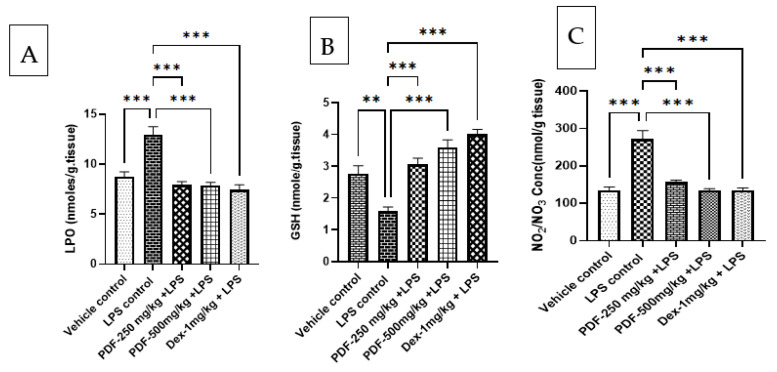
Effect of *Phoenix dactylifera* fruit extracts on oxidative and nitrative stress parameters in lipopolysaccharide treated rats, (**A**) Lipid peroxidation, (**B**) Reduced glutathione and (**C**) Nitric oxide metabolite. Numerical values are represented as mean ± SEM (n = 6), ** *p* < 0.01 and *** *p* < 0.001.

The administration of LPS significantly elevated the levels of LPO (*p* < 0.001) and nitrite (*p* < 0.001) and reduced GSH (*p* < 0.01) in the rat brain tissue when compared to the vehicle control group. Treatment with both doses of PDF to LPS-treated rats restored the levels of LPO (*p* < 0.001), nitrite (*p* < 0.001) and GSH (*p* < 0.001) when compared to the LPS-control rats. These results are comparable with the dexamethasone (*p* < 0.001) treated rats. The effect of PDF on oxidative and nitrative stress in LPS-treated rats is shown in Figure 6. 

#### 2.5.2. Effect of PDF on TNF-α and IL-6 Levels

**Figure 7:**
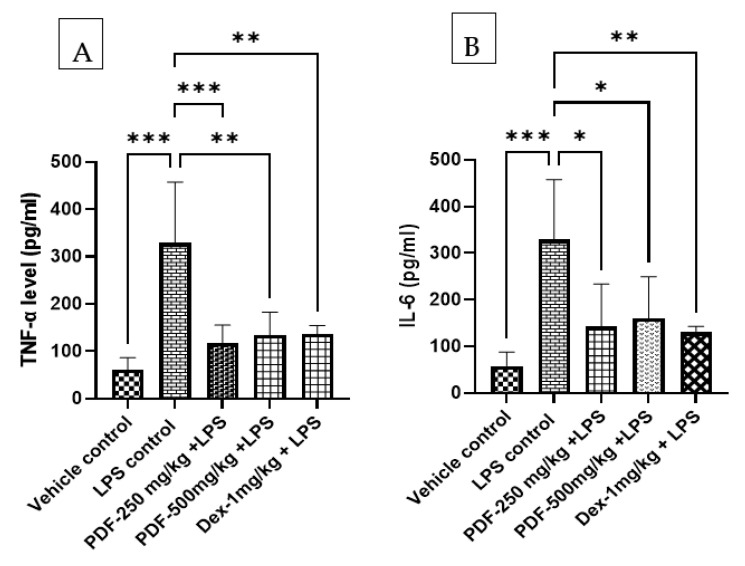
Effect of *Phoenix dactylifera* fruit extracts on (**A**) Tumor necrosis factor α and (**B**) Interleukin-6 in lipopolysaccharide treated rats. Numerical values are represented as mean ± SEM (n = 6), * *p* < 0.05, ** *p* < 0.01 and *** *p* < 0.001.

The inflammatory markers TNF-α and IL-6 levels were significantly (*p* < 0.001) elevated in the brain tissue homogenate when compared to the vehicle control rats. The administration of PDF to LPS-treated rats reduced the levels of TNF-α and IL-6. The values of PDF at the dose of 250 mg/kg and 500 mg/kg found statistically significant TNF-α (*p* < 0.001 and *p* < 0.01) and IL-6 (*p* < 0.05 and *p* < 0.05) when compared to the LPS-control rats. Dexamethasone treatment also significantly (*p* < 0.01) reduced the levels of TNF-α and IL-6 when compared to the LPS control rats. The results of the effect of PDF on TNF-α and IL-6 levels are shown in Figure 7. 

## 3. Discussion

In sickness behaviour, the soluble proteins secreted at the site of infection or injury make endocrine, autonomic, and behaviour changes in the victims [17,18,19]. The soluble proteins, interleukin (IL)-1, IL-6, and tumour necrosis factor-α (TNF-α) are essential pro-inflammatory cytokines that are released by activated immune cells such as macrophages and dendritic cells [9,17]. Controlling the immune system and coordinating cell-mediated immune responses depends heavily on the secreted soluble proteins [17]. 

Pro-inflammatory cytokines connect with the brain to alter behaviour in addition to controlling the localised inflammation [18,19]. Moreover, it stimulates the brain to release additional pro-inflammatory cytokines [18,19,20]. Mounting evidence suggests the involvement of pro-inflammatory cytokines IL-1, IL-6 and TNF-α in sickness behaviour [21,22]. Data from the current study convincingly confirm the aforementioned conclusions, showing that the brain tissue of LPS control mice has higher concentrations of IL-6 and TNF-α. These findings are consistent with the previously published reports on LPS-induced sickness behaviour in animals [22,23,24]. PDF attenuated IL-6 and TNF-α levels in LPS-treated rats. Moreover, a decrease in food and water intake is one of the primary signs of immune system activation, which is the result of raised cytokine levels [23]. These conclusions are supported by the findings of the current investigation. There was a drastic reduction in water and food intake in the LPS-treated rats compared to the vehicle control. PDF treatment restored the water and food intake in the LPS-treated rats. This may be due to reduced cytokine levels in the treated rats. 

The brain creates significant quantities of peroxides and reactive oxygen species (ROS) as a result of a rapid inflammatory response to LPS [25,26]. The production of ROS is the primary cause of oxidative stress and is a significant risk factor for a variety of neuropsychiatric diseases [22]. In the present study, increased levels of MDA and nitrite and reduced levels of GSH in the LPS-treated animals support the above findings. Both doses of PDF reduced the restored GSH levels and attenuated the increased levels of MDA and nitrite in the LPS-treated rat brain. These results support the previously published research data on the antioxidant properties of Ajwa dates [7,8]. 

Reduced mobility and depressed behaviours are the characteristic features of LPS-induced illness behaviour [22]. Administration of LPS resulted in decreased locomotor activities, altered despair behaviour and increased immobility in tail suspension test in rats. Treatment with PDF at both the tested doses attenuated the behavioural alteration in the LPS-treated rats. These results support the protective effect of PDF against LPS-induced behavioural changes in animals. These results are in agreement with the recent study on the neuroprotective activities of Ajwa seed extract in toxin-induced neuronal insults in animals [27]. 

LPS is also known to cause hepatic injury in rodents, hepatocytes contain the liver enzymes AST and ALT, which are released from the hepatocytes when the liver is injured [28]. It was discovered that abnormalities in cerebral neurotransmission that lead to sickness behaviours are connected to inflammatory liver injury [29,30]. In line with the aforementioned findings, higher levels of ALT and AST were seen in the serum of LPS-treated rats in the current investigation. Nevertheless, giving PDF to LPS-treated rats reduced the levels of ALT and AST in the treated rat serum, showing that PDF had a protective effect against the hepatic damage that LPS had caused in the rats. This demonstrates how PDF protected the rats from LPS-induced sickness behaviour.

According to the behavioural and biochemical parameters of the current study findings, PDF protects rats from LPS-induced sickness behaviour. It was evident by its attenuation of behavioural changes, restoration of antioxidant levels, reduction of oxidative and nitrative stress, reduction of proinflammatory cytokines, restoration of water and food intake and attenuation of ALT and AST in LPS-treated animals. These findings are in agreement with research data published on Ajwa dates as neuroprotective and beneficial in memory improvement in neuro-compromised animals [27,31]. 

The standardisation of PDF revealed that it has a considerable amount of total phenolic and flavonoid content and quercetin. The positive effects of PDF on LPS-induced sickness behaviour in rats could be attributed to these active ingredients.

## 4. Material and Methods

### 4.1. Drug and Chemicals 

Ajwa dates were purchased from the local market of Al Dawadmi, Kingdom of Saudi Arabia. Lipopolysaccharide (Product No-L2630, CAS No. 93572-42-0) and 5′-dithiobis-2-nitrobenzoic acid (Product No-D218200 CAS Number: 69-78-3) (Sigma–Aldrich, St. Louis, MI, USA), dexamethasone (Cadila Healthcare Ltd., Ahmedabad, India). Trichloroacetic acid and thiobarbituric acid were purchased from Loba Chemical Pvt. Ltd. in Mumbai, India. The remaining substances, including solvents, were all of the analytical grade.

### 4.2. Animals

Male adult Wistar rats (200–250 g) were purchased from King Abdul Aziz University’s, Faculty of Pharmacy’s Animal Centre, Jeddah, Saudi Arabia. The rats were housed on a 12-h light/dark cycle at 26 °C, and unlimited access to water and food. All animals were handled and cared for according to the guidelines of research on living organisms and its regulations and the rules governing ethics of scientific research at the University of Shaqra, Kingdom of Saudi Arabia. The Scientific Research Ethics Committee (ERC SU 20220071) of Shaqra University in Shaqra, Saudi Arabia, gave its approval to the study.

### 4.3. Extract Preparation 

The fruits of Ajwa date were stripped of their pits, allowed to dry at room temperature, and then powdered in a stainless-steel blender. The powder of Ajwa date fruits was extracted with methanol and water (4:1, *v*/*v*) for 5 h using an orbital shaker. The obtained extract was filtered and centrifuged for 10 min at 4000 g. Then the supernatant was concentrated at 40 °C under reduced pressure for 3 h using a rotary evaporator. The crude extract of Ajwa date fruits (PDF) was stored in a freezer in a dark glass bottle until its use.

### 4.4. Standardisation of PDF

#### 4.4.1. Total Phenolic Content and Flavonoid Content Determination

Total phenolics were determined in extract using the Folin-Ciocalteu method [32]. The amount of phenolic contents in each gram of extract was represented as mg gallic acid. The AlCl_3_ method was used for the determination of the total flavonoid content in the extracts [33]. The amount of flavonoid in each gramme of extract was represented as mg quercetin. 

#### 4.4.2. Estimation of Quercetin Using HPLC

The HPLC chemo profiling of methanolic extract of Ajwa date was carried out using the Shimadzu HPLC system (Kyoto, Japan) equipped with dual pump LC-20AD binary system, photodiode array (PDA) detector SPD-M20A, RP C_18_ column (5 µm, 4.6 × 250 mm). Standard quercetin (10 mg) was taken in a 50 mL volumetric flask, dissolved, and diluted (standard stock). Further, 5.0 mL of the above solution was diluted to 10 mL with diluent. Exactly 10.54 mg of methanolic extract of Ajwa date was weighed, taken in a 50 mL volumetric flask (0.2108 mg/mL), dissolved and diluted to volume with diluent and filtered through a 0.45-micron syringe filter.

Isocratic elution was performed using 70% methanol, 30% milli-q water and 0.1% trifluoroacetic acid. The flow rate and the injection volume were set at 1.0 mL/min and 20 µL, respectively. The chromatograms at 254 nm were analysed and compared.

### 4.5. Experimental Grouping and Treatment 

The male adult Wistar rats were weighed and randomised into five groups containing six animals each (n = 6). Group-1 (vehicle control) and Group-2 (LPS control) were orally administered with distilled water. Group-3 and Group-4 were orally treated with PDF (250 and 500 mg/kg, respectively) and Group-5 was injected with dexamethasone (1 mg/kg, i.p.). All these treatments were given daily for 14 days.

On the 14th day 1 h after the above-mentioned treatment animals from Group-1 were intraperitoneally injected with normal saline and Groups 2–5 were challenged with 1 mg/kg of LPS (i.p.).

After 24 h of saline or LPS injection, animal food, and water consumption was monitored. The behavioural parameters such as locomotor activity, tail suspension test, and forced swim test, were conducted. Blood samples were collected by retroorbital plexus and used for the estimation of ALT and AST levels. Following a cervical dislocation, rats were killed and their complete brains were collected for biochemical analysis.

### 4.6. Behavioural Parameters 

#### 4.6.1. Locomotor Activity Scores

The locomotor activity scores of the animals were recorded as per the method described by Shaikh et al. (2016). To record the locomotor activity scores each animal was separately placed in the activity cage for 5 min and the score displayed on the box was recorded [23]. 

#### 4.6.2. Tail Suspension Test 

In the tail suspension test, individual rats were hung 50 cm from the ground for 6 min from the tip of their tails using adhesive tape. Immobility time was measured during the last 5 min by a video camera and recorded in seconds [34].

#### 4.6.3. Despair Behaviour Test 

The despair behaviour of the rats was measured by the forced swim test. Rats were individually placed into the open cylindrical transparent plastic container (diameter 30 cm, height 50 cm) filled with water (25–27 °C) to a depth of 30 cm. Rats were made to swim for 6 min, then were returned to their plastic cages. The duration of immobility during the test was videotaped as the absence of movement required to keep the head above the water or climb up against the wall during the last five min [35]. 

### 4.7. Estimation of ALT and AST Levels in Serum

The blood samples were centrifuged at 4500 rpm for 15 min, serum was separated and used for the estimation of ALT and AST by using commercially available diagnostic kits (Quimica Clinica Aplicada S.A. QCA, Tarragona, Spain).

### 4.8. Biochemical Estimations in Brain Tissue

#### 4.8.1. Tissue Preparation

Brain tissues were dissected, and the hippocampus was separated on an ice-cold surface. Using a tissue homogenizer, a 10% homogenate was prepared in 0.1 M of ice-cold, pH 7.4 phosphate-buffered saline, and centrifuged at 10,000 rpm for 15 min at 4 °C [36,37]. The resulting supernatant was used to estimate biochemical parameters such as lipid peroxidation (LPO), reduced glutathione (GSH), nitrite, IL-6 and TNF-α.

#### 4.8.2. Oxidative and Nitrative Stress Parameters

Lipid peroxide formation was analysed by measuring the thiobarbituric-acid reactive substances in the form of malondialdehyde (MDA) content in the homogenate. The MDA contents are expressed in nmoles/g tissue [38]. GSH levels were determined by Ellman (1959) method and expressed as mmol/mg of tissue [39]. The nitrite content in the supernatant was estimated using Griess reagent by recording the absorbance at 540 nm using a microplate reader (Biotek, Shoreline, WA, USA). The nitrite levels are expressed in nmol/g tissue.

#### 4.8.3. Estimation of IL-6 and TNF-α Levels

TNF-α and IL-6 were estimated using specific ELISA kits (GENLISA, Rat IL-6 ELISA Cat: KB3068, Lot No RI60921; GENLISA, Rat TNF-α ELISA Cat: KB3145, Lot No RTA0122; (Krishgen BioSystems, Mombai, India) as per the instructions of the kit manufacturer. The absorbance is recorded at 450 nm using a microplate reader (Biotek, USA).

### 4.9. Statistical Analyses

Graphpad Prism 8.0 (Graphpad Software Inc., Boston, MA, USA) was used for all Statistical analyses. All data were represented as mean ± standard error of the mean. One-way ANOVA followed by Tukey’s test was used for analysis and *p* < 0.05 was considered statistically significant.

## 5. Conclusions

The results of the current study suggest that Ajwa dates have a protective effect against LPS-induced sickness behaviour in rats. Ajwa dates are thought to have health benefits because of their anti-inflammatory and antioxidant properties, and the presence of active ingredients such as quercetin. To clarify its molecular mechanism and validate its effects on human patients, more research is necessary.

## Figures and Tables

**Table 1:** Effect of PDF on water and food intake in LPS treated rats.

Parameters	Vehicle Control	LPS Control	PDF-250 mg/kg + LPS	PDF-500 mg/kg + LPS	Dex-1 mg/kg + LPS
Water intake (mL/6 h)	18.0 ± 0.60	6.52 ± 0.57	12.0 ± 0.46	15.3 ± 0.56	15.6 ± 0.59
Food intake (g/6 h)	6.00 ± 0.57	2.66 ± 0.16	4.50 ± 0.34	4.66 ± 0.33	4.83 ± 0.40

All the values are expressed in mean ± SEM. (n = 6). Where, LPS—lipopolysaccharide, PDF—*Phoenix dactylifera* fruit extracts and Dex—dexamethasone.

## Data Availability

All data generated or analyzed during this study are included in this published article.
